# Practical Therapeutics

**Published:** 1871-11

**Authors:** 


*Practical Therapeutics.* By Edward John Warring, M. D., F. L. S. Second American, from the Third London Edition. Philadelphia: Lindsay & Blakeston, 1871.

The appearance of a Second Edition of this work upon Therapeutics at once attracted our attention as likely to have undergone modification, and to contain considerable material not hitherto incorporated in any standard work. Glancing at the preface, we find in the author's own words, the changes and the omissions, which have been made. He says:

" In common with all other branches of knowledge, Therapeutics has within the last few years, made rapid strides, and the facts brought to light have been of a very diversified and important character. A notice of these in the following pages was manifestly indispensable, but to have engrafted them *en masse* on the matter contained in the previous editions was considered unadvisable, as by so doing the size of the volume would have been greatly increased without any commensurate benefit. Such a course, indeed, would have been attended with positive disadvantage, as the results of modern inquiry have tended in many instances to modify or overthrow views previously entertained; so that, in addition to increased bulk, itself objectionable, the volume would have presented in juxtaposition statements and counter-statements which could not well have failed to have been a source of considerable embarrassment to the stndent and young practitioner.

Under these circumstances it appeared expedient to rewrite the work, which has accordingly been done to a great extent, and several modifications have been introduced; some of the articles, *e, g.*, Antimony, Calomel, and Blood-letting, have been considerably abridged, several unofficinal articles of minor importance have been omitted altogether, the farmulæ have been arranged in a more condensed form, the foot-note references have been diminished in number or incorporated with the text, and by the introduction of more numerous synonyms in the body of the work the necessity for the Index of Medicines, extending over fourteen pages, has been obviated. By these means space has been rendered available for new matter, and advantage has been taken of it to introduce notices of Chloral, Bromide of Mercury, Iodide of Methyl, Bichloride of Methylene, Protoxide of Nitrogen, Sandalwood Oil, Apomorphia, and other new remedies, together with extended notices of other medicines, which, though not strictly new, have acquired increased importance from their claims as therapeutic agents, having only of late years been fully recognized; such, for example, as Bromide of Potassium, Calabar Bean, Carbolic and Sulphurous Acids, Permanganate of Potash, and the Alkaline Hypophosphites and Hyposulphites. In Part II space has been found for articles on the Hypodermic and Endermine methods of treatment, and to the article Antidotes has been appended a brief sketch of the most approved methods of treatment in cases of poisoning.

Notwithstanding these and other additions, this Edition, as compared with the last, shows a decrease in the number of pages, but it is confidently believed that though reduced in bulk it will not be found to have lost any portion of its practical utility, the object invariably aimed at throughout the compilation of the following pages."

In watching the progress or change in therapeutics, we are struck with wonder and admiration—wonder at the rapid change that is constantly taking place as to the value of medicinal substances, and admiration for the honesty and liberality of men in so readily and thoroughly correcting their previous opinions when convinced that they were wrong. Looking over the Chapter on Calomel, which formerly required many pages to detail the diseases in which it was essential as a curative agent we find the same space is now required to speak of the diseases in which it was formerly employed, but is now wholly abandoned. The same of Antimony, Bleeding, Leeching, Cupping, Blistering, &c., &c., remedies so long in vogue by at least all Englishmen.

This work in its present edition is a positive contribution to our standard therapeutics, and we most earnestly recommend it to the perusal of all who have held to former opinions, reluctantly changing their views, to correspond with the more careful observations which have established the therapeutics of the present time.

